# 2-[(5-Chloro-2-hy­droxy­benzyl­idene)amino]-3′,6′-bis­(diethyl­amino)­spiro­[isoindoline-1,9′-xanthen]-3-one

**DOI:** 10.1107/S1600536810019549

**Published:** 2010-05-29

**Authors:** Zhi-hong Xu, Wei-yun Guo, Bo-wei Su, Xu-Ke Shen, Feng-ling Yang

**Affiliations:** a College of Chemistry and Chemical Engineering, Xuchang University, Xuchang, Henan Province 461000, People’s Republic of China

## Abstract

The title compound, C_35_H_35_ClN_4_O_3_, resulted from a spiro­lactam ring closure of rhodamine B dye. The xanthene ring system is approximately planar [r.m.s. deviation = 0.050 (9) Å for the xanthene ring]. The dihedral angles formed by the spiro­lactam and 5-chloro-2-hy­droxy­benzene rings with the xanthene ring system are 87.9 (7) and 79.1 (7)°, respectively.

## Related literature

For rhodamine derivatives bearing a lactam unit, see: Deng *et al.* (2009 ▶); Kwon *et al.*, 2005 ▶; Tian & Peng (2008 ▶); Wu *et al.* (2007 ▶); Xu *et al.* (2009 ▶); Zhang *et al.* (2008 ▶).
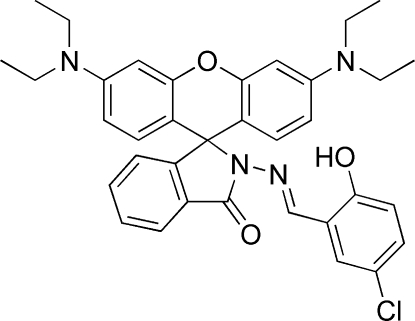


## Experimental

### 

#### Crystal data

C_35_H_35_ClN_4_O_3_
*M* *_r_* = 595.12
Orthorhombic, 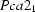

*a* = 21.609 (9) Å
*b* = 11.892 (5) Å
*c* = 12.355 (5) Å
*V* = 3175 (2) Å^3^
*Z* = 4
Mo *K*α radiation
μ = 0.16 mm^−1^
*T* = 296 K
0.35 × 0.32 × 0.29 mm

#### Data collection

Bruker APEXII CCD diffractometer
Absorption correction: multi-scan (*SADABS*; Bruker, 2005 ▶) *T*_min_ = 0.946, *T*_max_ = 0.955
16073 measured reflections
5528 independent reflections
2820 reflections with *I* > 2σ(*I*)
*R*_int_ = 0.079

#### Refinement

*R*[*F* ^2^ > 2σ(*F* ^2^)] = 0.049
*wR*(*F* ^2^) = 0.088
*S* = 1.02
5528 reflections
394 parameters
1 restraint
H-atom parameters constrained
Δρ_max_ = 0.20 e Å^−3^
Δρ_min_ = −0.25 e Å^−3^
Absolute structure: Flack (1983 ▶), 2521 Friedel pairs
Flack parameter: −0.05 (8)

### 

Data collection: *APEX2* (Bruker, 2005 ▶); cell refinement: *SAINT* (Bruker, 2005 ▶); data reduction: *SAINT*; program(s) used to solve structure: *SHELXS97* (Sheldrick, 2008 ▶); program(s) used to refine structure: *SHELXL97* (Sheldrick, 2008 ▶); molecular graphics: *SHELXTL* (Sheldrick, 2008 ▶); software used to prepare material for publication: *SHELXTL*.

## Supplementary Material

Crystal structure: contains datablocks I, global. DOI: 10.1107/S1600536810019549/cs2127sup1.cif

Structure factors: contains datablocks I. DOI: 10.1107/S1600536810019549/cs2127Isup2.hkl

Additional supplementary materials:  crystallographic information; 3D view; checkCIF report

## Figures and Tables

**Fig. 1.**
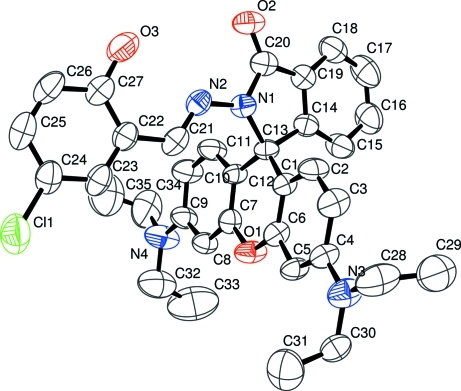
The molecular structure of the title compound, with H atoms omitted and with displacement ellipsoids drawn at 50% probability level

## supplementary crystallographic information

### Comment

Among many fluorescent compounds, rhodamine dyes are known to have excellent photophysical properties, and they are one of the most widely used fluorophores for labeling and sensing biomolecules. There are a few single-crystal reports on rhodamine derivatives bearing a lactam moiety (Xu *et al.*, 2009; Kwon *et al.*, 2005; Wu *et al.*, 2007; Zhang *et al.*, 2008; Tian *et al.*, 2008; Deng *et al.*, 2009). Detailed information on their molecular and crystal structures is necessary to understand their photophysical and photochemical properties.

In agreement with other reported models, (Xu *et al.*, 2009; Wu *et al.*, 2007; Zhang *et al.*, 2008; Tian *et al.*, 2008) the main skeleton of the molecule is formed by the xanthene ring and the spirolactam-ring. As shown in Figure 1, the atoms of the xanthene ring or the spirolactam-ring are both nearly planar and are almost perpendicular to each other. The dihedral angle between the xanthene and the spirolactam ring fragment mean planes is 87.9 (7)°. The dihedral angle between the xanthene mean plane and the 2-hydroxy-5-cholorobenzene ring is 79.1 (7)°.

### Experimental

A portion of rhodamine B hydrazide (0.46 g, 1 mmol) and 2-hydroxy-5-chlorobenzaldehyde (0.17 g, 1.1 mmol) were mixed in 20 ml ethanol to which three drops acetic acid was added. The reaction solution was refluxed for 3 hours under N_2_ atmosphere, the precipatate was separated and washed by ethanol to give 0.30 g of the title compound in 50% yield. Single crystals suitable for X-ray measurements were obtained from the mother liquid by slow solvent evaporation at room temperatures.

### Refinement

The H atoms attached to C, N and O atoms were placed in geometrically calculated positions (C—H = 0.93–0.97 Å and O—H = 0.82 Å) and refined as riding, with *U*_iso_(H) = 1.2*U*_eq_(C, N) or 1.5*U*_eq_(methyl C, O).

### Crystal data

**Table:**

C_35_H_35_ClN_4_O_3_	*F*(000) = 1256
*M**_r_* = 595.12	*D*_x_ = 1.245 Mg m^−^^3^
Orthorhombic, *P**c**a*2_1_	Mo *K*α radiation, λ = 0.71073 Å
Hall symbol: P 2c -2ac	Cell parameters from 1377 reflections
*a* = 21.609 (9) Å	θ = 2.5–17.6°
*b* = 11.892 (5) Å	µ = 0.16 mm^−^^1^
*c* = 12.355 (5) Å	*T* = 296 K
*V* = 3175 (2) Å^3^	Block, red
*Z* = 4	0.35 × 0.32 × 0.29 mm

### Data collection

**Table:**

Bruker APEXII CCD diffractometer	5528 independent reflections
Radiation source: fine-focus sealed tube	2820 reflections with *I* > 2σ(*I*)
graphite	*R*_int_ = 0.079
φ and ω scans	θ_max_ = 25.2°, θ_min_ = 1.7°
Absorption correction: multi-scan (*SADABS*; Bruker, 2005)	*h* = −21→25
*T*_min_ = 0.946, *T*_max_ = 0.955	*k* = −14→14
16073 measured reflections	*l* = −14→14

### Refinement

**Table:**

Refinement on *F*^2^	Hydrogen site location: inferred from neighbouring sites
Least-squares matrix: full	H-atom parameters constrained
*R*[*F*^2^ > 2σ(*F*^2^)] = 0.049	*w* = 1/[σ^2^(*F*_o_^2^) + (0.020*P*)^2^ + 0.0114*P*] where *P* = (*F*_o_^2^ + 2*F*_c_^2^)/3
*wR*(*F*^2^) = 0.088	(Δ/σ)_max_ = 0.001
*S* = 1.01	Δρ_max_ = 0.20 e Å^−^^3^
5528 reflections	Δρ_min_ = −0.25 e Å^−^^3^
394 parameters	Extinction correction: *SHELXL97* (Sheldrick, 2008), Fc^*^=kFc[1+0.001xFc^2^λ^3^/sin(2θ)]^-1/4^
1 restraint	Extinction coefficient: 0.0018 (2)
Primary atom site location: structure-invariant direct methods	Absolute structure: Flack (1983), 2521 Friedel pairs
Secondary atom site location: difference Fourier map	Flack parameter: −0.05 (8)

### Special details

**Table:**

Geometry. All esds (except the esd in the dihedral angle between two l.s. planes) are estimated using the full covariance matrix. The cell esds are taken into account individually in the estimation of esds in distances, angles and torsion angles; correlations between esds in cell parameters are only used when they are defined by crystal symmetry. An approximate (isotropic) treatment of cell esds is used for estimating esds involving l.s. planes.
Refinement. Refinement of *F*^2^ against ALL reflections. The weighted *R*-factor wR and goodness of fit *S* are based on *F*^2^, conventional *R*-factors *R* are based on *F*, with *F* set to zero for negative *F*^2^. The threshold expression of *F*^2^ > σ(*F*^2^) is used only for calculating *R*-factors(gt) etc. and is not relevant to the choice of reflections for refinement. *R*-factors based on *F*^2^ are statistically about twice as large as those based on *F*, and *R*- factors based on ALL data will be even larger.

### Fractional atomic coordinates and isotropic or equivalent isotropic displacement parameters (Å^2^)

**Table:**

	*x*	*y*	*z*	*U*_iso_*/*U*_eq_	
C1	0.91042 (15)	0.2527 (3)	0.2707 (3)	0.0444 (10)	
C2	0.92493 (16)	0.3631 (3)	0.2392 (3)	0.0556 (11)	
H2	0.9307	0.4173	0.2926	0.067*	
C3	0.93088 (18)	0.3942 (3)	0.1323 (3)	0.0610 (12)	
H3	0.9403	0.4684	0.1151	0.073*	
C4	0.92276 (16)	0.3146 (3)	0.0482 (3)	0.0514 (10)	
C5	0.90881 (15)	0.2049 (3)	0.0793 (3)	0.0498 (10)	
H5	0.9034	0.1499	0.0265	0.060*	
C6	0.90281 (15)	0.1763 (3)	0.1872 (3)	0.0480 (10)	
C7	0.88582 (16)	0.0256 (3)	0.3112 (3)	0.0487 (10)	
C8	0.87418 (16)	−0.0896 (3)	0.3190 (3)	0.0504 (11)	
H8	0.8687	−0.1325	0.2568	0.060*	
C9	0.87086 (18)	−0.1398 (3)	0.4202 (3)	0.0581 (11)	
C10	0.88058 (19)	−0.0710 (3)	0.5111 (3)	0.0709 (13)	
H10	0.8796	−0.1027	0.5799	0.085*	
C11	0.89161 (17)	0.0435 (3)	0.4998 (3)	0.0627 (12)	
H11	0.8975	0.0870	0.5615	0.075*	
C12	0.89403 (16)	0.0949 (3)	0.3985 (3)	0.0435 (9)	
C13	0.90153 (15)	0.2201 (3)	0.3879 (3)	0.0433 (9)	
C14	0.84695 (17)	0.2814 (3)	0.4418 (3)	0.0469 (10)	
C15	0.78553 (18)	0.2786 (3)	0.4104 (4)	0.0660 (12)	
H15	0.7734	0.2405	0.3482	0.079*	
C16	0.7425 (2)	0.3343 (4)	0.4747 (4)	0.0814 (15)	
H16	0.7009	0.3328	0.4556	0.098*	
C17	0.7606 (2)	0.3919 (4)	0.5666 (4)	0.0858 (15)	
H17	0.7311	0.4284	0.6085	0.103*	
C18	0.8217 (2)	0.3960 (3)	0.5970 (4)	0.0689 (12)	
H18	0.8340	0.4341	0.6591	0.083*	
C19	0.86485 (18)	0.3410 (3)	0.5316 (3)	0.0489 (10)	
C20	0.93194 (19)	0.3317 (3)	0.5431 (3)	0.0514 (10)	
C21	1.03453 (16)	0.1615 (3)	0.3910 (3)	0.0563 (11)	
H21	1.0103	0.1392	0.3326	0.068*	
C22	1.09773 (17)	0.1217 (3)	0.4016 (3)	0.0524 (10)	
C23	1.12309 (18)	0.0528 (3)	0.3199 (3)	0.0580 (11)	
H23	1.0995	0.0352	0.2593	0.070*	
C24	1.1822 (2)	0.0114 (3)	0.3287 (4)	0.0641 (12)	
C25	1.21808 (19)	0.0326 (4)	0.4194 (4)	0.0769 (13)	
H25	1.2574	0.0015	0.4259	0.092*	
C26	1.1946 (2)	0.1004 (4)	0.4997 (4)	0.0796 (15)	
H26	1.2186	0.1161	0.5604	0.096*	
C27	1.1352 (2)	0.1458 (4)	0.4911 (3)	0.0609 (12)	
C28	0.9453 (2)	0.4583 (4)	−0.0927 (4)	0.0861 (15)	
H28A	0.9664	0.4545	−0.1619	0.103*	
H28B	0.9740	0.4894	−0.0403	0.103*	
C29	0.8901 (3)	0.5355 (4)	−0.1026 (4)	0.1127 (19)	
H29A	0.8574	0.4974	−0.1404	0.169*	
H29B	0.9018	0.6018	−0.1419	0.169*	
H29C	0.8760	0.5563	−0.0317	0.169*	
C30	0.91860 (19)	0.2621 (4)	−0.1446 (3)	0.0635 (12)	
H30A	0.9042	0.3014	−0.2086	0.076*	
H30B	0.8863	0.2103	−0.1227	0.076*	
C31	0.9757 (2)	0.1955 (4)	−0.1738 (4)	0.1004 (17)	
H31A	1.0088	0.2461	−0.1914	0.151*	
H31B	0.9669	0.1484	−0.2350	0.151*	
H31C	0.9877	0.1495	−0.1134	0.151*	
C32	0.8484 (3)	−0.3280 (4)	0.3399 (4)	0.109 (2)	
H32A	0.8608	−0.4034	0.3605	0.131*	
H32B	0.8748	−0.3041	0.2807	0.131*	
C33	0.7837 (3)	−0.3307 (5)	0.3019 (5)	0.177 (3)	
H33A	0.7571	−0.3534	0.3603	0.266*	
H33B	0.7799	−0.3834	0.2434	0.266*	
H33C	0.7718	−0.2572	0.2774	0.266*	
C34	0.8455 (3)	−0.3014 (4)	0.5475 (6)	0.125 (2)	
H34A	0.8259	−0.2458	0.5935	0.150*	
H34B	0.8193	−0.3676	0.5438	0.150*	
C35	0.9045 (3)	−0.3290 (5)	0.5864 (6)	0.150 (3)	
H35A	0.9210	−0.3904	0.5450	0.225*	
H35B	0.9017	−0.3505	0.6611	0.225*	
H35C	0.9313	−0.2650	0.5797	0.225*	
Cl1	1.21296 (5)	−0.07129 (11)	0.22454 (12)	0.0958 (4)	
N1	0.95241 (13)	0.2637 (2)	0.4589 (3)	0.0472 (8)	
N2	1.01262 (14)	0.2276 (2)	0.4631 (3)	0.0489 (8)	
N3	0.92790 (14)	0.3435 (3)	−0.0588 (3)	0.0665 (10)	
N4	0.85785 (18)	−0.2527 (3)	0.4319 (3)	0.0830 (12)	
O1	0.88834 (11)	0.0645 (2)	0.2056 (2)	0.0611 (7)	
O2	0.96649 (13)	0.3715 (2)	0.6133 (2)	0.0667 (8)	
O3	1.11542 (13)	0.2139 (3)	0.5725 (2)	0.0799 (9)	
H3A	1.0809	0.2383	0.5579	0.120*	

### Atomic displacement parameters (Å^2^)

**Table:**

	*U*^11^	*U*^22^	*U*^33^	*U*^12^	*U*^13^	*U*^23^
C1	0.046 (2)	0.046 (2)	0.040 (3)	−0.0038 (18)	−0.0038 (18)	0.000 (2)
C2	0.067 (3)	0.050 (3)	0.049 (3)	−0.015 (2)	−0.005 (2)	−0.009 (2)
C3	0.075 (3)	0.053 (3)	0.055 (3)	−0.022 (2)	−0.003 (2)	0.013 (2)
C4	0.058 (3)	0.057 (3)	0.040 (3)	−0.011 (2)	−0.006 (2)	0.003 (2)
C5	0.067 (3)	0.052 (3)	0.030 (2)	−0.008 (2)	−0.001 (2)	0.004 (2)
C6	0.052 (2)	0.046 (3)	0.046 (3)	−0.0112 (19)	−0.004 (2)	0.008 (2)
C7	0.049 (2)	0.049 (3)	0.048 (3)	0.000 (2)	0.002 (2)	0.006 (2)
C8	0.063 (3)	0.042 (3)	0.046 (3)	−0.0063 (19)	0.001 (2)	−0.004 (2)
C9	0.087 (3)	0.039 (2)	0.048 (3)	−0.004 (2)	0.002 (2)	0.000 (2)
C10	0.120 (4)	0.052 (3)	0.041 (3)	−0.009 (3)	0.002 (3)	0.009 (2)
C11	0.094 (3)	0.053 (3)	0.041 (3)	−0.003 (2)	0.001 (2)	−0.006 (2)
C12	0.054 (2)	0.046 (2)	0.031 (2)	−0.0047 (19)	0.0000 (19)	−0.001 (2)
C13	0.044 (2)	0.043 (2)	0.043 (2)	−0.0087 (18)	−0.0043 (19)	−0.007 (2)
C14	0.048 (2)	0.042 (2)	0.051 (3)	−0.0081 (19)	0.001 (2)	−0.002 (2)
C15	0.061 (3)	0.065 (3)	0.072 (3)	−0.002 (2)	0.004 (3)	−0.019 (3)
C16	0.054 (3)	0.083 (3)	0.108 (5)	0.009 (3)	0.004 (3)	−0.011 (4)
C17	0.068 (4)	0.080 (4)	0.110 (5)	0.011 (3)	0.027 (3)	−0.015 (3)
C18	0.085 (3)	0.059 (3)	0.063 (3)	0.005 (3)	0.008 (3)	−0.006 (2)
C19	0.051 (3)	0.048 (3)	0.048 (3)	−0.003 (2)	0.006 (2)	0.003 (2)
C20	0.069 (3)	0.045 (3)	0.040 (3)	−0.009 (2)	0.005 (2)	0.007 (2)
C21	0.046 (3)	0.069 (3)	0.053 (3)	−0.008 (2)	−0.007 (2)	0.012 (2)
C22	0.043 (2)	0.059 (3)	0.055 (3)	−0.004 (2)	−0.002 (2)	0.009 (2)
C23	0.048 (3)	0.075 (3)	0.050 (3)	−0.001 (2)	−0.003 (2)	0.021 (2)
C24	0.054 (3)	0.083 (3)	0.055 (3)	0.011 (2)	0.006 (3)	0.017 (2)
C25	0.052 (3)	0.093 (4)	0.086 (4)	0.008 (3)	−0.012 (3)	0.020 (3)
C26	0.052 (3)	0.110 (4)	0.077 (4)	−0.006 (3)	−0.023 (3)	0.003 (3)
C27	0.055 (3)	0.073 (3)	0.055 (3)	−0.005 (2)	−0.006 (3)	0.009 (3)
C28	0.121 (4)	0.087 (4)	0.050 (3)	−0.045 (3)	−0.001 (3)	0.018 (3)
C29	0.185 (6)	0.057 (3)	0.096 (4)	−0.014 (4)	−0.037 (4)	0.013 (3)
C30	0.081 (3)	0.075 (3)	0.034 (2)	0.003 (3)	0.000 (2)	0.011 (2)
C31	0.091 (4)	0.115 (4)	0.096 (4)	0.013 (3)	0.020 (3)	0.013 (3)
C32	0.186 (7)	0.061 (3)	0.081 (4)	−0.019 (4)	0.003 (4)	0.003 (3)
C33	0.195 (8)	0.181 (6)	0.155 (7)	−0.090 (6)	−0.022 (6)	−0.032 (6)
C34	0.137 (5)	0.049 (3)	0.190 (7)	−0.019 (3)	−0.056 (5)	0.022 (4)
C35	0.153 (6)	0.115 (5)	0.184 (8)	−0.001 (4)	0.000 (5)	0.034 (5)
Cl1	0.0795 (8)	0.1268 (10)	0.0810 (9)	0.0372 (7)	0.0101 (8)	0.0157 (9)
N1	0.0449 (19)	0.053 (2)	0.044 (2)	−0.0050 (16)	0.0026 (17)	−0.0028 (17)
N2	0.046 (2)	0.058 (2)	0.043 (2)	−0.0033 (16)	−0.0016 (17)	0.0066 (18)
N3	0.088 (3)	0.063 (2)	0.048 (2)	−0.0181 (19)	−0.001 (2)	0.013 (2)
N4	0.155 (4)	0.044 (2)	0.050 (3)	−0.014 (2)	0.013 (2)	0.006 (2)
O1	0.101 (2)	0.0459 (16)	0.0368 (17)	−0.0196 (15)	−0.0012 (16)	0.0044 (14)
O2	0.084 (2)	0.0645 (19)	0.0511 (19)	−0.0140 (15)	−0.0083 (16)	−0.0106 (16)
O3	0.078 (2)	0.092 (2)	0.069 (2)	−0.0026 (18)	−0.0253 (19)	0.000 (2)

### Geometric parameters (Å, °)

**Table:**

C1—C6	1.384 (4)	C22—C27	1.400 (5)
C1—C2	1.405 (4)	C22—C23	1.411 (5)
C1—C13	1.512 (5)	C23—C24	1.374 (5)
C2—C3	1.377 (5)	C23—H23	0.9300
C2—H2	0.9300	C24—C25	1.386 (5)
C3—C4	1.416 (5)	C24—Cl1	1.751 (4)
C3—H3	0.9300	C25—C26	1.375 (5)
C4—N3	1.372 (5)	C25—H25	0.9300
C4—C5	1.393 (5)	C26—C27	1.398 (5)
C5—C6	1.382 (5)	C26—H26	0.9300
C5—H5	0.9300	C27—O3	1.359 (4)
C6—O1	1.384 (4)	C28—N3	1.477 (5)
C7—C12	1.370 (5)	C28—C29	1.511 (5)
C7—O1	1.385 (4)	C28—H28A	0.9700
C7—C8	1.396 (5)	C28—H28B	0.9700
C8—C9	1.387 (5)	C29—H29A	0.9600
C8—H8	0.9300	C29—H29B	0.9600
C9—N4	1.379 (4)	C29—H29C	0.9600
C9—C10	1.405 (5)	C30—N3	1.448 (5)
C10—C11	1.390 (5)	C30—C31	1.510 (5)
C10—H10	0.9300	C30—H30A	0.9700
C11—C12	1.394 (5)	C30—H30B	0.9700
C11—H11	0.9300	C31—H31A	0.9600
C12—C13	1.503 (4)	C31—H31B	0.9600
C13—N1	1.499 (4)	C31—H31C	0.9600
C13—C14	1.539 (5)	C32—N4	1.461 (5)
C14—C19	1.372 (5)	C32—C33	1.477 (6)
C14—C15	1.383 (4)	C32—H32A	0.9700
C15—C16	1.391 (5)	C32—H32B	0.9700
C15—H15	0.9300	C33—H33A	0.9600
C16—C17	1.383 (6)	C33—H33B	0.9600
C16—H16	0.9300	C33—H33C	0.9600
C17—C18	1.373 (5)	C34—C35	1.402 (6)
C17—H17	0.9300	C34—N4	1.565 (7)
C18—C19	1.396 (5)	C34—H34A	0.9700
C18—H18	0.9300	C34—H34B	0.9700
C19—C20	1.461 (5)	C35—H35A	0.9600
C20—O2	1.239 (4)	C35—H35B	0.9600
C20—N1	1.390 (4)	C35—H35C	0.9600
C21—N2	1.279 (4)	N1—N2	1.371 (4)
C21—C22	1.451 (5)	O3—H3A	0.8200
C21—H21	0.9300		
			
C6—C1—C2	115.7 (3)	C23—C24—C25	121.3 (4)
C6—C1—C13	122.0 (3)	C23—C24—Cl1	119.7 (4)
C2—C1—C13	122.2 (3)	C25—C24—Cl1	119.0 (4)
C3—C2—C1	122.5 (4)	C26—C25—C24	119.0 (4)
C3—C2—H2	118.8	C26—C25—H25	120.5
C1—C2—H2	118.8	C24—C25—H25	120.5
C2—C3—C4	120.8 (3)	C25—C26—C27	120.7 (4)
C2—C3—H3	119.6	C25—C26—H26	119.7
C4—C3—H3	119.6	C27—C26—H26	119.7
N3—C4—C5	121.2 (4)	O3—C27—C26	117.5 (4)
N3—C4—C3	122.0 (4)	O3—C27—C22	121.7 (4)
C5—C4—C3	116.8 (4)	C26—C27—C22	120.8 (4)
C6—C5—C4	121.1 (4)	N3—C28—C29	112.5 (4)
C6—C5—H5	119.5	N3—C28—H28A	109.1
C4—C5—H5	119.5	C29—C28—H28A	109.1
C5—C6—C1	123.1 (3)	N3—C28—H28B	109.1
C5—C6—O1	114.5 (3)	C29—C28—H28B	109.1
C1—C6—O1	122.3 (3)	H28A—C28—H28B	107.8
C12—C7—O1	122.4 (3)	C28—C29—H29A	109.5
C12—C7—C8	124.0 (4)	C28—C29—H29B	109.5
O1—C7—C8	113.7 (3)	H29A—C29—H29B	109.5
C9—C8—C7	119.6 (4)	C28—C29—H29C	109.5
C9—C8—H8	120.2	H29A—C29—H29C	109.5
C7—C8—H8	120.2	H29B—C29—H29C	109.5
N4—C9—C8	121.6 (4)	N3—C30—C31	114.3 (4)
N4—C9—C10	120.9 (4)	N3—C30—H30A	108.7
C8—C9—C10	117.5 (3)	C31—C30—H30A	108.7
C11—C10—C9	121.1 (4)	N3—C30—H30B	108.7
C11—C10—H10	119.5	C31—C30—H30B	108.7
C9—C10—H10	119.5	H30A—C30—H30B	107.6
C10—C11—C12	121.7 (4)	C30—C31—H31A	109.5
C10—C11—H11	119.1	C30—C31—H31B	109.5
C12—C11—H11	119.1	H31A—C31—H31B	109.5
C7—C12—C11	116.0 (3)	C30—C31—H31C	109.5
C7—C12—C13	122.8 (3)	H31A—C31—H31C	109.5
C11—C12—C13	121.1 (3)	H31B—C31—H31C	109.5
N1—C13—C12	111.8 (3)	N4—C32—C33	113.1 (5)
N1—C13—C1	112.2 (3)	N4—C32—H32A	109.0
C12—C13—C1	110.6 (3)	C33—C32—H32A	109.0
N1—C13—C14	98.3 (3)	N4—C32—H32B	109.0
C12—C13—C14	110.4 (3)	C33—C32—H32B	109.0
C1—C13—C14	113.0 (3)	H32A—C32—H32B	107.8
C19—C14—C15	120.7 (4)	C32—C33—H33A	109.5
C19—C14—C13	112.3 (3)	C32—C33—H33B	109.5
C15—C14—C13	127.0 (4)	H33A—C33—H33B	109.5
C14—C15—C16	118.0 (4)	C32—C33—H33C	109.5
C14—C15—H15	121.0	H33A—C33—H33C	109.5
C16—C15—H15	121.0	H33B—C33—H33C	109.5
C17—C16—C15	121.0 (4)	C35—C34—N4	104.2 (5)
C17—C16—H16	119.5	C35—C34—H34A	110.9
C15—C16—H16	119.5	N4—C34—H34A	110.9
C18—C17—C16	121.0 (4)	C35—C34—H34B	110.9
C18—C17—H17	119.5	N4—C34—H34B	110.9
C16—C17—H17	119.5	H34A—C34—H34B	108.9
C17—C18—C19	117.8 (4)	C34—C35—H35A	109.5
C17—C18—H18	121.1	C34—C35—H35B	109.5
C19—C18—H18	121.1	H35A—C35—H35B	109.5
C14—C19—C18	121.5 (4)	C34—C35—H35C	109.5
C14—C19—C20	108.6 (4)	H35A—C35—H35C	109.5
C18—C19—C20	129.9 (4)	H35B—C35—H35C	109.5
O2—C20—N1	123.7 (4)	N2—N1—C20	117.1 (3)
O2—C20—C19	129.6 (4)	N2—N1—C13	127.6 (3)
N1—C20—C19	106.7 (4)	C20—N1—C13	113.9 (3)
N2—C21—C22	119.0 (4)	C21—N2—N1	121.1 (3)
N2—C21—H21	120.5	C4—N3—C30	121.8 (3)
C22—C21—H21	120.5	C4—N3—C28	121.7 (4)
C27—C22—C23	117.4 (4)	C30—N3—C28	116.5 (3)
C27—C22—C21	123.3 (4)	C9—N4—C32	122.9 (4)
C23—C22—C21	119.3 (4)	C9—N4—C34	119.4 (4)
C24—C23—C22	120.8 (4)	C32—N4—C34	117.3 (3)
C24—C23—H23	119.6	C6—O1—C7	119.0 (3)
C22—C23—H23	119.6	C27—O3—H3A	109.5
			
C6—C1—C2—C3	0.4 (6)	C17—C18—C19—C20	179.6 (4)
C13—C1—C2—C3	−177.6 (4)	C14—C19—C20—O2	−179.1 (4)
C1—C2—C3—C4	−0.3 (6)	C18—C19—C20—O2	−0.7 (7)
C2—C3—C4—N3	179.4 (4)	C14—C19—C20—N1	−1.1 (4)
C2—C3—C4—C5	−0.2 (6)	C18—C19—C20—N1	177.4 (4)
N3—C4—C5—C6	−179.1 (3)	N2—C21—C22—C27	4.5 (5)
C3—C4—C5—C6	0.5 (5)	N2—C21—C22—C23	−177.2 (3)
C4—C5—C6—C1	−0.4 (6)	C27—C22—C23—C24	0.1 (6)
C4—C5—C6—O1	179.5 (3)	C21—C22—C23—C24	−178.3 (3)
C2—C1—C6—C5	0.0 (5)	C22—C23—C24—C25	2.2 (6)
C13—C1—C6—C5	178.0 (3)	C22—C23—C24—Cl1	−178.6 (3)
C2—C1—C6—O1	−179.9 (3)	C23—C24—C25—C26	−2.7 (7)
C13—C1—C6—O1	−1.9 (5)	Cl1—C24—C25—C26	178.1 (3)
C12—C7—C8—C9	−0.5 (6)	C24—C25—C26—C27	1.0 (7)
O1—C7—C8—C9	179.6 (3)	C25—C26—C27—O3	−178.7 (4)
C7—C8—C9—N4	178.3 (4)	C25—C26—C27—C22	1.2 (6)
C7—C8—C9—C10	−1.0 (5)	C23—C22—C27—O3	178.2 (3)
N4—C9—C10—C11	−177.8 (4)	C21—C22—C27—O3	−3.5 (6)
C8—C9—C10—C11	1.5 (6)	C23—C22—C27—C26	−1.7 (6)
C9—C10—C11—C12	−0.5 (6)	C21—C22—C27—C26	176.6 (4)
O1—C7—C12—C11	−178.6 (3)	O2—C20—N1—N2	8.8 (5)
C8—C7—C12—C11	1.5 (5)	C19—C20—N1—N2	−169.4 (3)
O1—C7—C12—C13	4.9 (5)	O2—C20—N1—C13	176.5 (3)
C8—C7—C12—C13	−175.1 (3)	C19—C20—N1—C13	−1.7 (4)
C10—C11—C12—C7	−0.9 (6)	C12—C13—N1—N2	53.6 (4)
C10—C11—C12—C13	175.6 (3)	C1—C13—N1—N2	−71.3 (4)
C7—C12—C13—N1	−136.2 (3)	C14—C13—N1—N2	169.6 (3)
C11—C12—C13—N1	47.5 (5)	C12—C13—N1—C20	−112.6 (3)
C7—C12—C13—C1	−10.4 (5)	C1—C13—N1—C20	122.5 (3)
C11—C12—C13—C1	173.3 (3)	C14—C13—N1—C20	3.4 (3)
C7—C12—C13—C14	115.4 (4)	C22—C21—N2—N1	−177.3 (3)
C11—C12—C13—C14	−60.9 (4)	C20—N1—N2—C21	177.5 (3)
C6—C1—C13—N1	134.4 (3)	C13—N1—N2—C21	11.7 (5)
C2—C1—C13—N1	−47.7 (4)	C5—C4—N3—C30	0.6 (6)
C6—C1—C13—C12	8.9 (4)	C3—C4—N3—C30	−179.0 (4)
C2—C1—C13—C12	−173.2 (3)	C5—C4—N3—C28	−177.3 (4)
C6—C1—C13—C14	−115.5 (4)	C3—C4—N3—C28	3.1 (6)
C2—C1—C13—C14	62.4 (4)	C31—C30—N3—C4	−86.1 (4)
N1—C13—C14—C19	−4.0 (4)	C31—C30—N3—C28	91.8 (4)
C12—C13—C14—C19	113.0 (3)	C29—C28—N3—C4	−87.4 (5)
C1—C13—C14—C19	−122.6 (3)	C29—C28—N3—C30	94.6 (5)
N1—C13—C14—C15	177.8 (4)	C8—C9—N4—C32	1.8 (7)
C12—C13—C14—C15	−65.2 (5)	C10—C9—N4—C32	−178.9 (4)
C1—C13—C14—C15	59.3 (5)	C8—C9—N4—C34	−171.5 (4)
C19—C14—C15—C16	−2.3 (6)	C10—C9—N4—C34	7.8 (6)
C13—C14—C15—C16	175.8 (3)	C33—C32—N4—C9	−87.6 (6)
C14—C15—C16—C17	0.7 (6)	C33—C32—N4—C34	85.8 (6)
C15—C16—C17—C18	0.1 (7)	C35—C34—N4—C9	−87.2 (6)
C16—C17—C18—C19	0.6 (7)	C35—C34—N4—C32	99.2 (5)
C15—C14—C19—C18	3.1 (6)	C5—C6—O1—C7	175.3 (3)
C13—C14—C19—C18	−175.2 (3)	C1—C6—O1—C7	−4.9 (5)
C15—C14—C19—C20	−178.3 (3)	C12—C7—O1—C6	3.4 (5)
C13—C14—C19—C20	3.4 (4)	C8—C7—O1—C6	−176.7 (3)
C17—C18—C19—C14	−2.2 (6)		
